# Situation models, mental simulations, and abstract concepts in discourse comprehension

**DOI:** 10.3758/s13423-015-0864-x

**Published:** 2015-06-19

**Authors:** Rolf A. Zwaan

**Affiliations:** Department of Psychology, Erasmus University Rotterdam, 3000 DR Rotterdam, The Netherlands

**Keywords:** Language comprehension, Embodied cognition, Mental models, Language/memory interactions, Abstract concepts

## Abstract

This article sets out to examine the role of symbolic and sensorimotor representations in discourse comprehension. It starts out with a review of the literature on situation models, showing how mental representations are constrained by linguistic and situational factors. These ideas are then extended to more explicitly include sensorimotor representations. Following Zwaan and Madden (2005), the author argues that sensorimotor and symbolic representations mutually constrain each other in discourse comprehension. These ideas are then developed further to propose two roles for abstract concepts in discourse comprehension. It is argued that they serve as pointers in memory, used (1) *cataphorically* to integrate upcoming information into a sensorimotor simulation, or (2) *anaphorically* integrate previously presented information into a sensorimotor simulation. In either case, the sensorimotor representation is a specific instantiation of the abstract concept.

An influential theoretical development in language comprehension research of the 1970s, 1980s, and 1990s was the introduction of the construct of a mental model or situation model (Bower & Morrow, 1990; Bransford, Barclay, & Franks, 1972; Glenberg, Meyer, & Lindem, 1987; Johnson-Laird, 1983; Kintsch, 1998; van Dijk & Kintsch, 1983; Zwaan & Radvansky, 1998). The basic idea behind situation models is that comprehension of a stretch of discourse involves the construction of a mental representation of the state of affairs denoted by that text rather than only a mental representation of the text itself. The computationally most explicit version of situation-model theory is Kintsch’s (1988; 1998) construction-integration model. It is an extension of Kintsch and van Dijk’s (1978) model of text recall. The key component of these models is the proposition. The mental representation of a text is conceptualized as a network of propositions that are linked via the arguments that they share. As Kintsch and van Dijk (1978) note, the proposition is “a convenient shorthand” for the mental representations that they hypothesized were formed during comprehension. In Kintsch’s (1988) construction-integration model, the proposition was the central representational unit, although Kintsch left the possibility open for other forms of mental representation, such as spatial representations (Kintsch, 1998).

## The event-indexing model

The event-indexing model (Zwaan, Langston, & Graesser, 1995; Zwaan, Magliano, & Graesser, 1995; Zwaan & Radvansky, 1998) is an attempt to specify relations among elements of a situation model, event representations. The basic unit of the situation model is an event representation. Events are thought to be related to one another on (at least) five dimensions: time, space, entity, causation, and motivation. As each incoming clause is processed, an event representation is formed and it is integrated with the event representation(s) currently in working memory based on its overlap with those representations on each of the five dimensions. If the event occurs within the same time frame as the events in working memory, there is overlap on the temporal dimension; if the event takes place within the same spatial region there is spatial overlap; if it involves the same entity (person or object), there is entity overlap; if it is causally related to the previous event(s) there is causal overlap; and if is part of the same goal/plan structure, there is motivational overlap.

Two simple assumptions are that these five dimensions are equally weighted and that their effects are additive. This leads to two predictions, one about online comprehension and one about the resulting mental representation in long-term memory. The more situational overlap the current event has with the contents of working memory, (1) the easier it should be to process the clause describing that event, and (2) the stronger the connections between the current event and the events in working memory should be in long-term memory. These predictions were supported in several empirical studies, as was the assumption that the effects of the dimensions were additive (e.g., Therriault, Rinck, & Zwaan, 2006; Zwaan, Radvansky, Hilliard, & Curiel, 1998; Zwaan, Langston, et al., 1995; Zwaan, Magliano, et al., 1995; see also Radvansky & Zacks, 2014). More recently, the event-indexing model has been extended into the domain of film understanding (Cutting & Iricinschi, 2015; Magliano, Miller, & Zwaan, 2001; Zacks, Speer, Swallow, & Maley, 2010).

## Addressing the grounding problem

In 1999 the present author submitted a manuscript on the event-indexing model. One of the reviewers was quite critical and remarked: “You’re talking about events and links, but the events are just empty nodes. What is IN those event nodes?” Unlike the present author at the time, the reader will recognize this as an articulation of the grounding problem (Harnad, 1990). Barsalou’s (1999) article on the perceptual symbol system proposed a way to address the grounding problem. However, while the theoretical richness of the article was impressive, empirical evidence for perceptual symbol systems was as yet forthcoming. Attempts to test predictions from perceptual symbol theory led to two early papers in which predictions from perceptual symbol theory were pitted against predictions from propositional theory, which reflected the mainstream of thinking about mental representation. Following Barsalou (1999), it was hypothesized that if a sentence implies, but does not explicitly mention, a perceptual characteristic of an entity, a purely propositional theory would not predict that that characteristic is represented, but perceptual symbol theory would. For example, the sentence “*The egg is in the carton*” does not explicitly state that the egg is whole. If people form visual representations of objects, however, then readers should represent the shape of a whole egg and it should be different when they read “*There was an egg in the skillet*.” Specifically, when asked whether a depicted object was mentioned in the just-read sentence, readers should respond more quickly to a picture of a whole egg than to one of one sunny side up after reading the first sentence, and vice versa for the second sentence. These predictions were supported in several experiments (Stanfield & Zwaan, 2001; Zwaan, Stanfield, & Yaxley, 2002; see Zwaan & Pecher, 2012, for direct replications). These two studies are only among the first in a large body of research. The past 15 years have provided a great deal of evidence to suggest that language comprehension involves sensorimotor representations (for a recent review of this literature, see Meteyard, Cuadrado, Bahrami, Vigliocco, 2012; for a meta-analysis of relevant behavioral findings, see Louwerse, Hutchinson, Tillman, & Recchia, 2015). Most of this work has used individual words or sentences as stimuli but recent neuroimaging research shows that sensorimotor representations are also activated during the comprehension of discourse (e.g., Chow, Mar, Xu, Liu, Wagage, & Braun, 2015; Kurby & Zacks, 2013; Nijhoff & Willems, 2015).

## The interplay between symbolic and sensorimotor representations

Does this evidence mean that linguistic factors have no role to play in grounded discourse comprehension? Certainly not. In the research on the event-indexing model, it was always assumed that textual relations (modeled as argument overlap) impact the comprehension process, and empirical evidence showed that they do. In the regression models that were used, situation model effects were always assessed after linguistic effects such as argument overlap had been taken into account. The evidence showed that situational overlap impacted comprehension even when textual relations were taken into account (Zwaan, Langston, et al., 1995; Zwaan, Magliano, et al., 1995). Continuing this line of thought, later work on grounded cognition has repeatedly made the point that linguistic factors (such as co-occurrence of linguistic constructions) play a role in comprehension (Taylor & Zwaan, 2009; Zwaan & Madden, 2005; Zwaan, 2004, 2008). As Zwaan and Madden (2005) noted: “This idea that associations between representations are formed through co-occurrence of linguistic constructions is central to [our] current theory.” Others have expressed similar views (Andrews, Frank, & Vigliocco, 2014; Barsalou, Santos, Simmons, & Wilson, 2008; Dove, 2011; Louwerse, 2011). One hypothesis is that linguistic and perceptual processes mutually constrain each other (Zwaan & Madden, 2005; Zwaan, 2008) for example, linguistic co-occurrence leads to predictions of upcoming linguistic constructions and of the associated perceptual representations, while perceptual simulations may lead to the prediction of upcoming perceptual aspects and the associated linguistic constructions.

## Abstract concepts in context

Thus far, this review has only focused on concrete information. This reflects in part that the author’s own empirical work has focused on concrete concepts. Most natural discourse contains a mixture of concrete and abstract concepts, however. Recent neuroimaging experiments have shown that the degree to which sensorimotor information is activated during sentence comprehension depends on the linguistic context. The same action verb (e.g., “cut”) will generate more activation in the (pre)motor cortex when read in a literal context than in a figurative context (Desai et al., 2011; Raposo, Moss, Stamatakis, & Tyler, 2009; Schuil, Smits, & Zwaan, 2013; but see Boulenger, Hauk, & Pulvermüller, 2009). This body of studies suggests that the degree to which sensorimotor representations are engaged during language comprehension is variable and a function of context (Lebois, Wilson-Mendenhall, & Barsalou, 2015; Louwerse, 2011; Taylor & Zwaan, 2009).

There is reason to assume that abstract concepts are more sensitive to contextual constraints than concrete concepts (Barsalou & Wiemer-Hastings, 2005; Schwanenflugel, 1991). For example, although concrete words are typically comprehended faster than abstract words, this difference disappears when supportive context is offered (Schwanenflugel & Shoben, 1983). A recent neuroimaging study showed that when abstract words are presented in an appropriate context, the same patterns of activation of semantically appropriate nonlinguistic content occur as with concrete words (Wilson-Mendenhall, Simmons, Martin, & Barsalou, 2013).

## The clutch metaphor

What is the mechanism that drives the activation of sensorimotor representations during language comprehension? Mahon (2015) introduces the helpful metaphor of a clutch to conceptualize how sensorimotor representations can be engaged to varying extents in language comprehension. A clutch is a device that connects a driving shaft (e.g., the motor in a power drill) to the driven shaft (e.g., the drill itself) and can do this to varying degrees, meaning that the torque of the motor gets transferred fully, partly, or not at all to the driven shaft. In Mahon’s clutch metaphor, symbolic processing is the driving shaft (doing all the initial processing work) and sensorimotor representations are the driven shaft, getting involved in the comprehension process to differing degrees but never driving the process. As Mahon notes, the clutch metaphor is useful in that it helps us understand a hallmark of human cognition in general and language use in particular, our ability to disengage ourselves from the here and now, which Hockett (1960) called displacement. However, Mahon’s assumption that symbolic processing is doing all the relevant work might be premature. The experiments that are used as evidence that symbolic representations precede sensorimotor ones typically investigate activation as a function of the presentation of a decontextualized linguistic stimulus – a word or a sentence. This likely biases findings toward the primacy of symbolic over sensorimotor representations. When all you have is a word, it makes sense that the first thing to come to mind is another word. But what if the word occurs in a discourse context, as is the case outside of psychology experiments (Graesser, Millis, & Zwaan, 1997)? Then there is a representation in working memory: the active part of the evolving situation model plus the associated linguistic structures (Ericsson & Kintsch, 1995; Sanford & Garrod, 1981; van Dijk & Kintsch, 1983; Zwaan, Radvansky, Hilliard, & Curiel, 1998). Research using the visual-world paradigm has provided a substantial body of evidence that nonlinguistic context has an immediate effect on word and sentence processing (e.g., Chambers, Tanenhaus, & Magnuson, 2004; Cooper, 1974; Tanenhaus, Spivey-Knowlton, Eberhard, & Sedivy, 1995).

It is therefore plausible to assume that as longer stretches of text are read, more and more sensorimotor representations will become activated, which will, in turn, activate associated lexical representations. In such cases, the flow of activation between symbolic and sensorimotor representations is likely bidirectional, as Zwaan and Madden (2005) already proposed, and the two layers of representation mutually constrain each other to produce fluency in the comprehension process (Zwaan, 2008).

Taking Mahon’s (2015) proposal one step further, the appropriate metaphor is that of a two-way clutch. Given the linguistic nature of the stimulus, initial activation will necessarily flow from symbolic representations to sensorimotor ones and the degree to which this will happen is due to a number of contextual factors. Once sufficient sensorimotor context has been accumulated, activation flows both ways, from the symbolic system to the sensorimotor system and vice versa. This does not deny the possibility that comprehending a stretch of discourse text comprehension sometimes may not involve detailed sensorimotor representations at all, for example because the comprehender has limited background knowledge of the topic of the discourse or because or she is not a proficient user of the language of the discourse. Comprehension is not an all-or-nothing process. It is fault-tolerant and may occur at different levels of depth (Taylor & Zwaan, 2009). In many instances, a shallow symbolic representation (the kind of processing many psychology experiments seem to invite) may be sufficient to get by. Taylor and Zwaan (2009) give the example of someone telling someone else about a high jumper jumping over a bar and injuring his neck. Here is a slight extension of it. Someone not proficient in English may not know the proper contextual meaning of “bar” and may also not know the meaning of “injure.” Still, that person may come away with the understanding that a male individual jumped over something and something happened to his neck. This level of understanding might be sufficient in many instances. Someone proficient in English but with no experience with sports may understand that a high jumper jumped over a (high jumping) bar and somehow injured his neck. Clearly this person understands more of the sentence and, in fact, this level of understanding is sufficient to answer a simple comprehension question about the sentence (How did he injure his neck? Answer: By jumping over a bar). Questions of this kind are often posed in psycholinguistic experiments. Someone who has actually seen high-jumping being performed will infer – for example, by activating dynamic visual representations – that the high-jumper presumably performed the Fosbury flop (going backwards over the bar) and landed on his neck, thus injuring it. This comprehender is able to understand the (correct) causal connection between the jump and the injury and so has achieved a deeper level of comprehension than the previous comprehender. Someone who has actually performed the Fosbury flop will in addition be able to activate relevant motor and somatosensory representations, thus leading to a yet deeper, first person, understanding of the sentence. This comprehender may “feel the jumper’s pain.” The point of this example is that such a deep level of understanding is often not necessary “to get by” in many situations.

Consider Bransford, Barclay, and Johnson’s (1972) well-known “washing clothes” scenario. The scenario describes a sequence of vague actions involving vague objects. Here is how it starts out: “*The procedure is actually quite simple. First you arrange things into different groups. Of course*, *one pile may be sufficient depending on how much there is to do*…” These sentences are not completely incomprehensible but the comprehension they afford is severely limited because the referents of most words are unknown. What are the *things* the text mentions and what is the *procedure*? All we know is that the things can be arranged in groups (meaning that they are not affixed to some surface) and that they can be piled up (meaning that they cannot be spheres or needle-shaped). However, once the story’s title (*Washing Clothes*) is provided, the veil of abstraction is lifted and sensorimotor representations can be activated. For example, “*pile of things*” can now be taken to refer to a pile of laundry, for which a visual representation can be activated as well as a motor representation of piling up laundry. There have been neuroimaging studies involving *Washing Clothes*-type stories (e.g., St. George et al. 1999; Maguire, Frith, & Morris, 1999) but these were concerned with coherence. I am not aware if the relevant experiment has been done but one can conceive of the following one in which there are two between-subjects conditions. In one condition, the subjects read the text without title and then are instructed to reread it with the title. In the second condition they read the text both times without the title. The prediction is that there will be more sensorimotor activation in the title condition than in the control condition. Recent neuroimaging evidence shows that personal experience with the narrated events strongly modulates interactions between higher- and lower-level areas within the visual and motor processing systems (Chow et al., 2015). Thus, perhaps the neural instantiation of the clutch is as a modulator of the retrieval and integration of visual and motor knowledge into the evolving situation model.

## Symbolic representations in discourse comprehension

This analysis points to a potential division of labor between symbolic and grounded representations in extended discourse. It is possible that insufficient context is initially provided for the activation of relevant sensorimotor representations, though probably mostly not in such an extreme a manner as in the *Washing Clothes* case. In such cases symbolic representations function as placeholders, pointers in working memory that are part of a network of semantic associations that will be fleshed out with sensorimotor representations once sufficient context has been accumulated. In an earlier paper, I provided an example of this (Zwaan, 2014), which is developed further here. When presented out of context (as in many a psychology experiment) the word “justice” may invoke a variety of representations, which differ both between subjects and within subjects across time (Barsalou & Wiemer-Hastings, 2005). Barsalou and Wiemer-Hastings theorize that abstract and concrete concepts are similar in that they are both representations of situations. The two differ in that concrete concepts have a focal entity that is more or less the same across situations, whereas in abstract concepts, the focus is more diffuse. I consider this the most promising account of abstract concepts that is available in the literature. But how does this play out in discourse?

Consider two cases, many instantiations of which can be found in newspapers and novels. In the first case, the abstract term is presented early on in the discourse without much prior context. Suppose a text starts out with the sentence “*Today justice was served*”. At this point, the word *justice* does not have a contextually relevant sensorimotor representation associated with it because it is not clear what the context is, although it is possible that a (positive) emotional representation is activated given that abstract words have been found to be more emotionally valenced than concrete words (Kousta et al., 2011). Let us further assume that readers adopt a wait-and-see strategy in cases where the contextual meaning of a word is unclear and will not commit to a specific sensorimotor simulation, as this might prove contextually inappropriate and recovery might be cognitively costly. In such a case the reader uses the symbolic representation as a placeholder in an active state in working memory (Ericsson & Kintsch, 1995), analogous to what presumably occurs in the Bransford et al. (1972) experiment. This representation is subsequently used by the comprehension system to integrate subsequent information so that a situated simulation can be formed. To borrow a linguistic term, the abstract concept is used *cataphorically*, referring to later content (Gernsbacher & Shroyer, 1989).

The second case is complementary to the first. A text describes a specific case and court decision and concludes with the statement “*Today justice was served*.” In this case, there is a specific situation model that is now being labeled with the word *justice*. It is not that the word *justice* gives meaning to the situation. Rather, its current meaning is instantiated by the situation.,^1^,^2^ This label now serves as a pointer in memory to a specific instantiation of justice (a sensorimotor simulation) that can be used in further reference to the situation, either in the same discourse (assuming the article extends beyond the labeling) or in additional discourse (e.g., a letter to the editor). To borrow another term from linguistics, the representation is used *anaphorically*, referring to previous content.

## Conclusion

In this article, the literature on situation models and mental simulations has been reviewed in an initial attempt to integrate the two. I have argued that symbolic representations and the associations between them interact with sensorimotor representations to achieve fluent discourse comprehension. What is proposed here is a dual role for abstract concepts in discourse comprehension, inspired by Barsalou and Wiemer-Hastings (2005). On this proposal, abstract concepts in extended discourse function as (1) representations in working memory that serve to integrate multimodal information in a mental simulation of a specific situation, or (2) as representations in memory that serve as pointers to previously formed situational representations such that these can be reactivated at later stages. It is likely that these cataphoric and anaphoric functions of abstract concepts are two ends of a continuum and can be operative simultaneously if an abstract concept is provided midway through the description of a situation.

Thus, on this account abstract concepts are weakly associated with a set of sensorimotor representations and their associated symbolic representations (e.g., words) that is more diverse than that of concrete concepts. They acquire – either cataphorically or anaphorically – a specific sensorimotor instantiation in a discourse context. When abstract concepts operate cataphorically, their role is to provide a focus to the comprehension process, much like pronouns have been shown to do (Gernsbacher & Shroyer, 1989). When they occur anaphorically, they function as pointers to situational instantiations. To return to Mahon’s (2015) clutch metaphor, when an abstract concept is used cataphorically, the sensorimotor system is abruptly disengaged when the abstract concept is introduced, only to be gradually engaged again so that sensorimotor information can be used to form a situated instantiation of the concept. When an abstract concept is used anaphorically, the sensorimotor system was engaged all along and is now associated with a symbolic representation that functions as a pointer in memory.

There are some obvious limitations to these proposals. First, they need to be fleshed out further. Second, they might be more relevant to some types of discourse (e.g., narratives) than to others (e.g., scientific papers). It would be useful to take level of situational embeddedness (Zwaan, 2014) into account for a more complete analysis. Third, the cataphoric and anaphoric roles are quite possibly not the only roles that abstract concepts play in discourse. Nevertheless, I hope that these proposals provide a way forward in theorizing about grounded discourse comprehension.

## Footnotes

1 The inspiration for this idea was a conversation the author had with Art Graesser and Daniel Casasanto.

2 A recent example is a column in *the Guardian* by noted lawyer Alan Dershowitz, which desribes a case and concludes with the word *justice*http://www.theguardian.com/commentisfree/2015/jan/01/serial-adnan-syed-exonerated-new-trial.
